# Analysis of Clinicopathological and Molecular Features of Microcystic, Elongated, and Fragmented Pattern Invasion in Endometrioid Endometrial Cancer

**DOI:** 10.3390/cancers16203555

**Published:** 2024-10-21

**Authors:** Xiaobo Zhang, Bo Han, Danhua Shen

**Affiliations:** Department of Pathology, Peking University People’s Hospital, Beijing 100044, China; zhangxiaobo0326@163.com (X.Z.); boh@sdu.edu.cn (B.H.)

**Keywords:** endometrioid endometrial cancer, MELF, molecular classification, prognosis

## Abstract

**Background**: Microcystic, elongated, and fragmented (MELF) invasion is a special invasion pattern in endometrioid endometrial cancer (EEC). This study aimed to investigate the clinical, pathological, and molecular features of the MELF pattern and its prognostic value in patients with EEC. **Materials and Methods**: The clinical and pathological data of 342 patients with EEC were retrospectively collected at Peking University People’s Hospital from January 2019 to December 2022. Some key clinicopathological features were evaluated, including the tumor grade, Federation of Gynecology and Obstetrics (FIGO) staging, cervical stromal involvement, lymph node status, and lymphatic vascular space infiltration (LVSI). Immunohistochemical staining and molecular tests were performed, and the relevant literature was reviewed. **Results**: The MELF pattern was more prevalent in low-grade EEC. A significant correlation was found between the MELF pattern and advanced FIGO staging, LVSI, the depth of myometrial invasion, cervical stromal involvement, and lymph node metastasis (LNM). The incidence of mismatch-repair-deficient (MMRd) proteins was much higher in the MELF group than in the no-MELF group. Molecular testing revealed that, after copy number—low (CNL), microsatellite instability—high (MSI-H) was the second-most frequent subtype in the MELF group. The recurrence risk did not significantly differ between the MELF and no-MELF groups, but the differences among the four molecular subtypes were statistically significant. However, the MELF group experienced a shorter recurrence time. Among the four molecular subtypes, the recurrence risk was the highest in the CNH subgroup, followed by the MSI-H subgroup. **Conclusions**: MELF is a special invasion pattern in EEC and is associated with distinct clinicopathological and molecular characteristics, including the latest 2023 FIGO staging. Further research is warranted to explore its implications for treatment strategies and patient outcomes.

## Simple Summary

The aim of this study was to report on the clinical, pathological, and molecular features of endometrioid endometrial cancer (EEC) with and without microcystic, elongated, and fragmented (MELF) infiltration and to evaluate the latest phase of the Federation of Gynecology and Obstetrics (FIGO) staging, updated in 2023. MELF is a special invasion pattern in EEC and is associated with certain clinicopathological features, such as 2023 FIGO staging, tumor grade, the presence of LVSI, LNM, and mismatch-repair deficiency (MMRd). LVSI, LNM, and MMRd were more frequent in the MELF group than in the no-MELF group, and the differences were statistically significant (*p* < 0.05). Molecular classification was also applicable to the MELF pattern. The recurrence risk was highest in the copy number—high (CNH) subgroup, followed by the microsatellite instability—high (MSI-H) group, whereas POLE and copy number—low (CNL) were associated with a relatively good prognosis in this cohort. This study is a large-cohort investigation and provides valuable information regarding molecular classification and updated 2023 FIGO staging for MELF, highlighting the importance of integrating molecular and traditional histopathological assessments for risk evaluation. This approach could enhance prognostic accuracy and facilitate tailored therapeutic strategies.

## 1. Introduction

Endometrial carcinoma is one of the most common gynecologic cancers, and its incidence has gradually increased in recent years. EEC is the most common histologic type of endometrial cancer, accounting for nearly 80% of cases, the majority of which are low-grade and low-stage, associated with an excellent prognosis. One of the most important prognostic factors in ECC is clinical staging. For endometrial cancer, myometrial invasion is an important parameter, as defined by the FIGO staging system [1]. However, there are several types of invasion patterns in EEC, including the pushing infiltrative type, adenoma adenomyosis-like type, malignum-like type, MELF type, etc. [2]. MELF was first found and briefly described by Lee et al. in 1994 [3] and then clearly defined by Murray et al. in 2003 [4]. The patterns of invasion in patients with EEC include the microcystic, elongated, and fragmented pattern. This is a special pattern of myoinvasion where the elongated, clustered and lined by flattened epithelial glands were found in the myometrium. It is associated with LVSI and easily overlooked LNM [5], which can lead to a poor prognosis. However, some researchers have argued that this morphological change might be a process of degeneration, which is usually accompanied by fibromyxoid stromal changes [6]. Others considered MELF infiltration to be a special type of tumor–stroma interaction in the uterine myometrium during tumor development [7], which is akin to epithelial–mesenchymal transformation (EMT), suggesting a complex role in tumor development.

In 2021, the National Comprehensive Cancer Network (NCCN) uploaded its guidelines for uterine neoplasms, which recommended molecular subtyping for EC diagnosis [8]. In addition, the European Society of Gynecologic Oncology (ESGO)/European Society for Radiotherapy and Oncology (ESTRO)/European Society of Pathology (ESP) guidelines for EC further incorporated molecular subtypes into the risk stratification system for guiding postoperative adjuvant therapies [9]. The 2020 WHO Classification of Female Genital Tumors highlighted a significant correlation between MELF infiltration and the microsatellite instability—high (MSI-H) subtype [10]. In the 2023 edition of the FIGO staging system, molecular typing was incorporated, underscoring its growing importance in clinical practice [1]. Some studies have shown respective molecular classifications in EEC with the MELF pattern and indicated that combining molecular classification and pathological features is very important for the risk assessment of EEC [11,12].

In short, there have been various comments made about MELF invasion. At present, the clinical significance of MELF changes in EEC is unclear, although there seems to be an association between the presence of LVSI and the possibility of an increased risk of LNM. In order to further study the clinicopathological and molecular features of MELF infiltration and speculate on its prognosis, we conducted a study involving EEC patients with and without MELF infiltration who were operated on and treated at the Peking University People’s Hospital from January 2019 to December 2022.

## 2. Materials and Methods

### 2.1. Ethics Statement and Data Collection

The present research was approved by Peking University People’s Hospital. Patients were informed that the resected specimens would be stored by the Pathology Department of Peking University People’s Hospital. A total of 342 cases diagnosed between January 2019 and December 2022 were retrospectively consecutively collected for this study from our institution. In all cases, all pathology slides were independently reviewed by two experienced gynecological pathologists (Zhang and Shen) following the 5th WHO Classification of Tumors of Female Reproductive Organs [10]. Since MELF-pattern invasion was identified from surgical specimens, it was difficult to know the status of MELF-pattern invasion before surgery. Thus, all 342 cases were surgical specimens. Of these, 335 cases underwent complete surgical staging, including the following: total hysterectomy, bilateral salpingo-oophorectomy, and/or pelvic sentinel node biopsy. In seven cases, only bilateral adnexal hysterectomy was performed without lymph node dissection. Lymph node metastases were evaluated, including micrometastases (single or small clusters of tumor cells between 0.2 and 2 mm in size) and macrometastases (clusters of tumor cells >2 mm in size). LVSI was categorized into two groups based on the number of positive slices: <5/slice and ≥5/slice.

### 2.2. Diagnostic Criteria of MELF

The following diagnostic criteria were used to confirm MELF infiltration:

1. Microcysts were present and lined with cells with abundant eosinophilic cytoplasm and a vaguely squamoid appearance or lined with flattened cells, often containing neutrophils and occasionally eosinophils in the lumens.

2. Elongated structures were present and lined with the same cell types. Inflammatory cells, notably neutrophils, appeared around the glands and within the glandular lumen.

3. There were often significant inflammatory and edematous fibromyxoid interstitial reactions around the glands and exfoliated cells. Clusters of detached cells or individual cells were lying in edematous or myxoid tissue.

MELF infiltration was diagnosed if one of the above criteria was met [13].

### 2.3. Immunohistochemical Analysis

Immunohistochemical (IHC) testing was performed on labeled cases. All cases were tested for ER, PR, mismatch-repair (MMR) proteins, p16, and p53. Formalin-fixed paraffin-embedded (FFPE) blocks were sectioned (4 microns) and stained with specific antibodies for ER (6F11, Leica, RTU, Shanghai, China), PR (16, Leica, RTU, Shanghai, China), p53 (D07, 1:50, Roche, Roche Benchmark Ultra, Shanghai, China), and p16 (E6H4, Roche, Roche Benchmark Ultra). IHC for MMR proteins (mutL homolog 1 [MLH1], mutShomolog 2 [MSH2], mutS homolog 6[MSH6], PMS1 homolog 2 [PMS2]) was performed using the following antibodies: MSH2 (RED2, 1:100, MXB, Fuzhou, China), MSH6 (EP49, 1:100, MXB, Fuzhou, China), PSM2 (EP51, 1:100, MXB, Fuzhou, China), and MLH1 (MX063, 1:50, MXB, Fuzhou, China). Cases were considered to show a stable immunophenotype (MMRþ) if any tumor cell nuclei showed positive staining and an unstable immunophenotype (MMR-D) if all tumor cell nuclei were negative [14]. Stromal/lymphocyte staining and non-neoplastic endometrial glands were used as internal controls. Brown–yellow staining indicated a positive result. Ki-67-, p53-, ER-, and PR-positive sites were located in the nucleus, and p16-positive sites were located in the nucleus and/or cytoplasm. For p53, the complete lack of nuclear staining, strong diffuse nuclear staining (>80% of tumor cell nuclei), or diffuse cytoplasmic staining was defined as a mutated pattern [15]. Focal weak positive or weak to intermediate p53 immunohistochemical staining was considered the wild-type pattern [16]. The staining of ER and PR was considered positive if the tumor cell nuclei were stained in at least 1% of the tumor cells. All immunohistochemical stains had been previously validated for clinical use.

### 2.4. Molecular Detection

Tissue specimens taken during surgery were analyzed using next-generation sequencing (NGS) technology for molecular classification. DNA was extracted from FFPE samples using the Magen FFPE DNA Kit (Magen, Guangzhou, China) and quantified using a Qubit fluorometer (Invitrogen, Shanghai, China). Library preparation involved 10 ng of genomic DNA from each sample using a RingCap amplicon-based panel (SpaceGen, Xiamen, China). Sequencing reads were generated on a MiSeq platform (Illumina, San Diego, CA, USA). The genes in this NGS panel included MLH1, MSH2, MSH6, PMS2, TP53, and POLE. MSI status was detected by the NGS panel, which included 7 MSI loci (BAT25, BAT26, CAT-25, MONO-27, NR-21, NR-24, and NR-27). Raw sequencing data were trimmed and aligned to the human hg19 reference genome using Trimmomatic (version 0.36) and BWA (version 0.7.17). Next, aligned reads were processed to call SNVs and small Indels using Pisces (version 5.2.9), followed by variant annotation using ANNOVAR (version 20180426). MSIsensor-pro (version 1.2.0) was used to identify MS-stable (MSS, no MSI markers), MSI-low (MSI-L, one MSI marker), and MSI-high (MSI-H, two or more MSI markers) tumors without matched normal samples.

### 2.5. Statistical Analysis

Statistical analyses were performed using the IBM SPSS Statistics Software (version 25.0). The association variables were compared using χ^2^ or Fisher’s exact test. All *p*-values were two-sided, and *p* < 0.05 was considered statistically significant. Progression-free survival (PFS) was defined as the interval between the date of pathologic diagnosis and the first confirmed recurrence of the tumor; if there was no recurrence, the end date used was the date of the endpoint. PFS curves were used for statistical analysis.

## 3. Results

### 3.1. Clinicopathological Findings

A total of 342 cases with complete clinical and pathological data were included in this study, and 200 cases underwent successful molecular testing. The general clinicopathological features were compared between the MELF (63 cases) and no-MELF infiltration EEC groups (279 cases). The median ages of patients with and without MELF infiltration were 55 years (46–76 years old) and 51 years (42–81 years old), respectively, showing a statistically significant difference. The patients in the MELF group were older.

**Figure 1:**
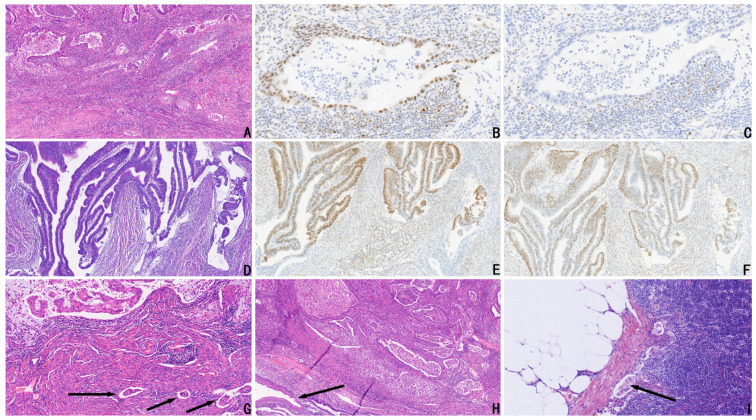
(**A**) Representative H&E image of EC with MELF (HE, ×100). (**B**) Corresponding image showing MLH1-positive expression (HE, ×200). (**C**) Corresponding image showing loss of PMS2, with positive expression seen in stromal cells (HE, ×200). (**D**) Representative H&E image of EC with MELF (HE, ×100). (**E**) Corresponding image showing scattered positive ER expression (HE, ×100). (**F**) Corresponding image showing scattered positive PR expression (HE, ×100). (**G**) Representative H&E image of EC with MELF and LVSI (marked with arrows). (**H**) Representative H&E image of EC with MELF and cervical stromal involvement (marked with arrows). (**I**) Lymph node metastasis (marked with arrows).

The proportions of pathological grades and FIGO stages in the two groups were different and were lower in the MELF group than in the no-MELF group. The positive rates of cervical stromal involvement, LVSI, and LNM were higher in the MELF group than in the no-MELF group, and the differences were statistically significant (*p* < 0.05). In the MELF group, substantial LVSI (≥5/slice) occurred in 11 cases, whereas in the no-MELF group, it occurred in 6 cases. Among the LNM cases, micro- and macrometastases occurred in eight and six cases in the MELF group and two and five cases in the no-MELF group, respectively. The infiltration depth of myometrial invasion was deeper in the MELF group than in the no-MELF group. Tumors were larger in the MELF group than in the no-MELF group (Table 1, Figure 1).

### 3.2. Molecular and IHC Findings

ER, PR, p16, and p53 protein immunoexpression patterns were analyzed. ER and PR showed different degrees of positive expression across all cases in the two groups. There were no significant differences in the ER and PR phenotypes between the two groups.

All cases showed a focal or patchy pattern of immunoexpression for p16. Only 3 cases with mutated p53 patterns were observed in the MELF group (3/63, 4.76%), whereas 20 such cases were seen in the no-MELF group (20/279, 7.17%); the other cases showed the wild-type p53 pattern. Mutated p53 expression was detected in 23 cases, with 22 cases positive for diffuse nuclear staining and 1 case completely negative, indicating aberrant/mutated p53. There were no significant differences in p16 and p53 phenotypes between the two groups (Table 2).

Regarding MMR proteins, 55 cases of MMRd were identified among the 342 cases: 20 cases in the MELF group (20/63, 31.75%) and 35 cases in the no-MELF group (35/279, 12.54%). The expression of MMR proteins was significantly different between the two groups, and the incidence rate of MMRd proteins was much higher in the MELF group. Our data showed that the loss of MLH1–PMS2 coexpression was the most frequent MMRd type in the two groups (11/63 and 22/279, respectively). The MMRd with the second-highest incidence was the loss of MSH2–MSH6 coexpression (3/63 and 9/279, respectively). The loss of only MLH1 expression occurred in two cases (1/63 and 1/279, respectively), the loss of only PSM2 expression occurred in four cases (2/63 and 2/279, respectively), and the loss of only MSH6 expression occurred in four cases (3/63 and 1/279, respectively) in the two groups (Table 2, Figure 1).

Genetic testing was performed in 200 cases, including 63 cases in the MELF group and 137 cases in the no-MELF group. The distribution of molecular subtypes was as follows: 61.90% (39/63) CNL, 28.57% (18/63) MSI-H, 4.76% (3/63) CNH, and 4.76% (3/63) POLE-mutated in the MELF group. In comparison, in the no-MELF group (n = 137), the distribution of molecular subtypes was 65.69% (90/137) CNL, 13.87% (19/137) MSI-H, 12.41% (17/137) CNH, and 8.03% (11/137) POLE-mutated. The CNL group was the most common among the four subtypes in both groups. Second to CNL, MSI-H was more common than the other two subtypes in both groups and was more frequent in the MELF group than in the no-MELF group. The difference between the two groups was statistically significant (*p* = 0.000).

**Figure 2:**
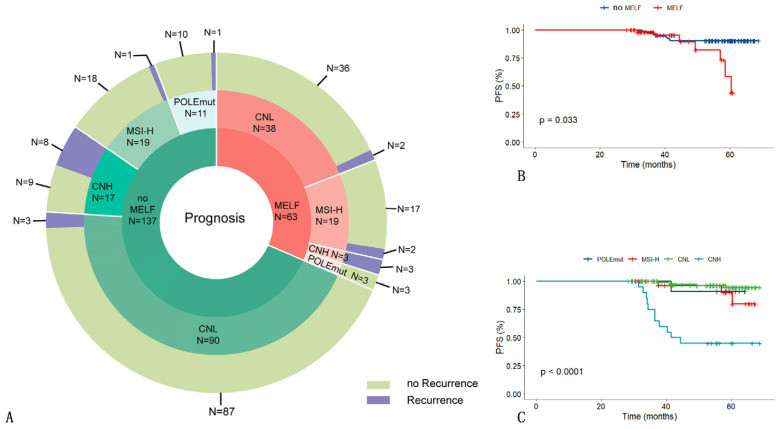
(**A**) Proportions of different outcomes for patients with different molecular subtypes in the MELF and no-MELF groups. (**B**) PFS curves in MELF and no-MELF groups in EEC. (**C**) PFS curves for 4 molecular subtypes in EEC. Note: MELF, microcystic, elongated, and fragmented; PFS, progression-free survival; POLEmuted, POLE mutation; MSI-H, microsatellite instability—high; CNL, copy number—low; CNH, copy number—high (including MELF and no-MELF groups).

PFS differences based on the four molecular subtypes of EEC were investigated in the two groups. The median follow-up time of EEC patients with the MELF pattern (63 cases) and without the MELF pattern (137 cases) was 43 months (range 18–60 months). During the follow-up period, 20 patients (20/200, 10.00%) suffered tumor recurrence, but not progression, and no patients died due to EEC during the follow-up period. Among the recurrence cases, 7 patients were in the MELF group, and 13 patients were in the no-MELF group; the recurrence risk was not different between the two groups (*p* = 0.72). However, the MELF group had a shorter recurrence time (*p* = 0.03). Eleven cases (5.50%) of CNH, five cases (2.50%) of CNL, three cases (1.50%) of MSI-H, and one case (0.50%) of POLEmuted experienced tumor recurrences in the two groups. Among the four molecular subgroups of EEC, the recurrence risk was the highest in the CNH subgroup, followed by MSI-H, whereas POLE and CNL were associated with a relatively good prognosis in this cohort (*p* = 0.02) (Figure 2).

## 4. Discussion

The MELF pattern is a special mode of myometrial invasion in EEC and has been reported with a highly variable frequency, ranging between 5.8% and 48% [17]. This significant fluctuation in frequency may be due to some studies including different pathological types of endometrial cancer. The frequency of the MELF pattern was 18.42% (63/342) in this study, which is lower than in some other reports. This may be because this study was retrospective in nature; the pathological features may not have been well understood at the initial diagnosis in some cases, and some cases may not have been accurately diagnosed by pathologists several years ago. In 1994, the morphological characteristics of a special infiltration pattern in EEC were initially recognized by Lee, Vacek, and Belinson [3]. They found that the EEC cells that infiltrated the myometrium became elongated, resembled endothelial cells in shape, and surrounded each other to form lumens. In 2003, the special morphological characteristics of myoinfiltration patterns in more than 100 cases of intrauterine carcinoma were analyzed by Murray et al., who first defined the term MELF [4], highlighting the microcystic, elongated, and fragmented pattern of invasion. Thereafter, many scholars carried out further research on the MELF invasion pattern. In 2020, in the WHO Classification of Female Genital Tumors, the concept of MELF invasion was first officially mentioned [10].

The 63 patients with MELF EEC in this study ranged in age from 46 to 76 years, and the 279 patients with no-MELF EEC ranged in age from 42 to 81 years. There was a significant difference in age between patients with and without MELF invasion (279 cases) (*p* < 0.01): the patients in the MELF group were older, so older EEC patients should be paid special attention. Research was also conducted on the correlations between MELF and adverse clinical parameters (older age, etc.) in EEC [18]. MELF invasion was more prevalent in low-grade EEC than in high-grade EEC, and its invasion pattern was very sparse [19]. The MELF pattern was frequently (11/12) identified in low-grade (FIGO grade 1 or 2) EEC, as previously reported. However, it has also been reported in the literature in high-grade (FIGO grade 3) EEC [20]. In this study, a higher proportion of MELF was found in low-grade EEC (61/63), with only two cases occurring in high-grade EEC (2/63). Some scholars have suggested that the MELF pattern is significantly associated with adverse histopathological features, such as large tumor size, deep myometrial invasion, papillary architecture, and mucinous differentiation, among patients with low-grade EEC [17,21]. Han et al. [22] found a significant correlation between the MELF pattern and cervical stromal involvement. In this study, the occurrence risk of larger tumor size, deep myometrial invasion, and cervical stromal involvement was higher in the MELF group than in the no-MELF group; these pathological parameters were different between the two groups, and the differences were statistically significant (*p* < 0.05). In contrast to patients without MELF infiltration, high probabilities of cervical stromal involvement and deep myometrial invasion are often reported in patients with the MELF pattern. Zhang et al. reported that [23] 50% (3/6) of cases with cervical stromal involvement displayed the MELF infiltrative growth pattern. Thus, our study provides another line of evidence that the MELF pattern might be associated with cervical stromal involvement, which contributes to an increase in FIGO staging (stage II). Nonetheless, no studies have addressed outcomes with regard to the association of MELF with lower or upper uterine segment disease, mainly because it does not affect FIGO staging, so scholars have not paid attention to it. In patients with the MELF pattern, extravaginal recurrence was observed to be more prevalent than vaginal recurrence or no recurrence, as reported in another study [24]. Because this study had a shorter follow-up time than the previous study, no cases of vaginal recurrence were found. Overall, these observations support the hypothesis that the MELF pattern is associated with more aggressive disease characteristics.

In addition, LVSI and LNM were reevaluated, and substantial LVSI was found in eight cases (≥5/slice). This led to an increase in FIGO staging because substantial LVSI (≥ 5/slice) is classified into FIGO stage II [1]. The 2023 FIGO staging system of EEC is the first version to consider the number of LVSIs as a parameter for staging. LVSI was one of the independent risk factors affecting the prognosis of patients with EEC. A large retrospective study identified LVSI as the strongest independent risk factor for LNM and overall survival, even in the absence of LNM, in patients with EEC [25]. Some studies have suggested that a higher incidence of LNM is observed with MELF invasion in low-grade EEC [17,19]. In this study, the positive rate of LNM in the MELF group was higher than in the no-MELF group (22.22% and 2.58%, respectively). In addition, lymph node status was assessed and divided into micrometastasis and macrometastasis. In the 2023 FIGO staging system, LNM still belongs to stage III. However, there are some minor changes for different metastasis patterns. Micrometastasis was much more frequent in the MELF group (eight cases); macrometastasis was observed in four cases. Some studies suggested that the prognosis of EEC patients in the MELF group was not much worse than that of patients in the no-MELF group and suggested that it might be related to lymph node micrometastasis because the prognosis for micrometastasis was relatively good. Thus, for MELF infiltration EEC, it is necessary to be careful in determining lymph node status, and immunohistochemical labeling of CKpan and CD68 should be performed when necessary to avoid missing isolated tumor cells and micrometastasis. This subcategorization also reflected the increasing utilization of sentinel lymph node ultrastaging, which enhanced the detection of small-volume diseases, including micrometastasis, ultimately improving patient management and outcomes. Furthermore, we also observed and compared the expression of different IHC markers in the two groups. The incidence of MMRd was higher in the MELF group than in the no-MELF group, corroborating previous research, including the WHO 2020 guidelines, which highlighted this association [10]. In this study, the coexpression loss rate of MLH1 and PMS2 proteins was the most frequent MMRd (55% and 62.86%, respectively). According to literature reports, germline mutations in MMR genes mainly resulted in MLH1–PMS2 coexpression loss, and mutations causing MSH2–MSH6 coexpression loss played a secondary role [26]. More MMRd cases were detected in cases with the MELF pattern, suggesting a potential connection to tumor immune reactions. It is necessary to further integrate the molecular mechanisms with the histologic morphology of the MELF pattern. Immune checkpoint blockade has emerged as an effective therapeutic strategy for some advanced cancers. Different expression patterns of IHC markers with MELF infiltration, such as the expression of ER and PR, were compared. The expression of ER and PR did not significantly differ between the two invasion patterns in EEC, but their expression was much lower in the MELF group than in the no-MELF group. This suggests that the MELF infiltration pattern might represent EMT and reflect a reversible change in tumor cell activity in the process of tumor invasion and progression. It is possible that this pattern is indicative of disrupted connections between the tumor cells in EMT, along with enhanced cell motility and easier invasion and migration, rather than representing a degenerative change. Some scholars have speculated that other genetic changes, such as elevated N-methyltransferase (NNMT), followed by the enhancement in tumor migration and invasion, might contribute to the characteristic morphology observed with the MELF pattern [27]. The expression of the tumor suppressor gene p16 was positive in MELF EEC. This might be related to the MELF pattern participating in tumor cell growth suppression or promoting tumor cell senescence through the activation of p53 and p16-RB channels [17]. However, in our study, the most common expression pattern of p53 was the wild type; only 23 cases had mutated patterns (3 cases with MELF, 20 cases with no MELF), and differences were not seen between the two groups. Differences in ER, PR, p53, and p16 expression were not detected between the two groups.

In 2013, The Cancer Genome Atlas (TCGA) project stratified endometrial cancer into four prognostic groups based on the gene mutation spectrum mutant: CNH, CNL, MSI-H, and POLEmuted [28]. This provided more accurate information for clinical treatment and prognosis. Therefore, it is of great significance to develop reliable methods in pathology laboratories so that TCGA stratification can be introduced into routine practice. A group of Chinese scholars found that integrating POLEmuted status with various clinicopathological factors, including staging, myometrial invasion (MI), and MELF, could assist in developing precise and individualized therapeutic strategies and improve the effectiveness of risk assessment for EEC [11]. In this study, molecular classification was performed using NGS detection. The results show that MELF-pattern (63 cases) and no-MELF-pattern EEC (137 cases) can be divided into four molecular subtypes, of which the most prevalent was the CNL subtype in both groups (61.90% and 65.69%, respectively). This result is similar to other literature reports [12] that indicated a higher prevalence of the CNL subtype among Chinese populations. After CNL, MSI-H was the subtype with the second-highest frequency (28.57% and 13.87%, respectively), and there was a significant difference between the two groups (*p* < 0.01): MSI-H was higher in the MELF group than in the no-MELF group. Some studies suggested that MELF might be associated with MMRd [29], which was consistent with the existing literature and the WHO 2020 classification, which identified MELF in MMRd EEC [10].

Because this study had a retrospective design, the follow-up period was relatively short (18–66 months), and the observation period was insufficient, the effect of the MELF pattern on prognosis was not fully evaluated. Most cases in this study were treated recently, and follow-ups were still ongoing. Among the 200 patients with molecular typing, no patients died due to EEC, although 20 patients (20/200, 10.00%) suffered tumor recurrence. According to our analysis, the recurrence risk did not differ between the two groups. The MELF group did not show a worse prognosis than the no-MELF group, but it had a shorter recurrence time. This might be associated with the shorter follow-up time in the no-MELF group. However, Qi argued that using the MELF pattern alone had limitations in predicting prognosis [30]. Among the four molecular subgroups of EEC, the CNH subtype had the highest recurrence risk and the poorest prognosis, followed by the MSI-H subtype, whereas the POLE and CNL subtypes were associated with a relatively good prognosis in this cohort.

## 5. Conclusions

In summary, MELF is a special invasion pattern in EEC and is associated with certain clinicopathological features, such as FIGO staging, tumor grade, and the presence of LVSI, LNM, and MMRd. Molecular classification was also applicable to the MELF pattern, highlighting the importance of integrating molecular and traditional histopathological assessments for risk evaluation. This approach could enhance prognostic accuracy and facilitate tailored therapeutic strategies. However, further larger, multi-center studies with extended follow-up periods are necessary to clarify the exact role of MELF in the prognosis and adjuvant therapy for EEC.

## Figures and Tables

**Table 1:** Primary clinicopathological characteristics of all patients.

	MELF (*n* = 63)	No MELF (*n* = 279)	*p*-Value
Age			0.000
≤50	5 (7.94%)	87 (31.18%)	
>50	58 (92.06%)	192 (68.82%)	
Tumor size			0.034
≤2 cm	13 (20.63%)	96 (34.41%)	
>2 cm	50 (79.37%)	183 (65.59%)	
Tumor grade			0.000
1	23 (36.51%)	159 (56.99%)	
2	38 (60.32%)	88 (31.54%)	
3	2 (3.17%)	32 (11.47%)	
2023 FIGO staging			0.000
I	31 (49.21%)	233 (83.51%)	
II	18 (28.57%)	25 (8.97%)	
III	14 (22.22%)	17 (6.09%)	
IV	0 (0%)	4 (1.43%)	
Myometrial invasion			0.000
<1/2	33 (52.38%)	233 (83.51%)	
≥1/2	30 (47.62%)	46 (16.49%)	
Cervical stromal involvement			0.003
Absent	50 (79.37%)	257 (92.11%)	
Present	13 (20.63%)	22 (7.89%)	
LNM			0.000
Negative	49 (77.78%)	265 (97.42%)	
positive	14 (22.22%)	7 (2.58%)	
Micrometastasis	8	2	
Macrometastasis	6	5	
LVSI			0.000
Absent	32 (50.79%)	254 (91.04%)	
Present	31 (49.21%)	25 (8.96%)	
<5/slice	20 (31.75%)	19 (6.81%)	
≥5/slice	11 (17.46%)	6 (2.15%)	

Note: MELF, microcystic, elongated, and fragmented; FIGO, International Federation of Gynecology and Obstetrics; LNM, lymph node metastasis; LVSI, lymphovascular space invasion.

**Table 2:** Primary immunohistochemical and molecular characteristics of all patients.

	MELF (*n* = 63)	No MELF (*n* = 279)	*p*-Value
ER			0.127
≤50%	23 (36.51%)	75 (26.88%)	
>50%	40 (63.49%)	204 (73.12%)	
PR			0.110
≤50%	20 (31.74%)	62 (22.22%)	
>50%	43 (68.26%)	217 (77.78%)	
p53			0.591
Wild type	60 (95.24%)	259 (92.83%)	
Abnormal	3 (4.76%)	20 (7.17%)	
P16			0.133
Patchy	63 (100.00%)	267 (95.70%)	
Diffuse	0 (0)	12 (4.30%)	
Immunophenotype MMR			0.001
Stable	43 (68.26%)	244 (87.46%)	
Unstable	20 (31.74%)	35 (12.54%)	
MLH1 and PMS2Abnormal	11 (55.00%)	22 (62.86%)	
MSH2 and MSH6 Abnormal	3 (15.00%)	9 (25.71%)	
MLH1 Abnormal	1 (5.00%)	1 (2.86%)	
PMS2 Abnormal	2 (10.00%)	2 (5.71%)	
MSH6 Abnormal	3 (15.00%)	1 (2.86%)	
Molecular classification			0.000
MSI-H	18 (28.57%)	19 (13.87%)	
CNL	39 (61.90%)	90 (65.69%)	
CNH	3 (4.76%)	17 (12.41%)	
POLEmuted	3(4.76%)	11(8.03%)	

Note: MELF, microcystic, elongated, and fragmented; POLEmuted, POLE mutation; MSI-H, microsatellite instability—high; CNL, copy number—low; CNH, copy number—high.

## Data Availability

The data presented in this study are available on request from the corresponding author.
